# Instantaneous, Dual-Frequency, Multi-GNSS Precise RTK Positioning Using Google Pixel 4 and Samsung Galaxy S20 Smartphones for Zero and Short Baselines

**DOI:** 10.3390/s21248318

**Published:** 2021-12-13

**Authors:** Chien Zheng Yong, Robert Odolinski, Safoora Zaminpardaz, Michael Moore, Eldar Rubinov, Jeremiah Er, Mike Denham

**Affiliations:** 1 National School of Surveying, University of Otago, 310 Castle Street, Dunedin 9016, New Zealand; chienzheng.yong@utm.my (C.Z.Y.); erje7160@student.otago.ac.nz (J.E.); mike.denham@otago.ac.nz (M.D.); 2 Faculty of Built Environment and Surveying, Universiti Teknologi Malaysia, Johor Bahru 81310, Malaysia; 3 School of Science, College of Science, Engineering and Health, RMIT University, GPO Box 2476V, Melbourne, VIC 3001, Australia; safoora.zaminpardaz@rmit.edu.au; 4 Geoscience Australia, GPO Box 378, Canberra, ACT 2601, Australia; mikemoorester@gmail.com; 5 FrontierSI, Goods Shed, Village Street, Docklands, VIC 3008, Australia; erubinov@frontiersi.com.au

**Keywords:** smartphone positioning, real-time kinematic (RTK), multi-GNSS, dual frequency

## Abstract

The recent development of the smartphone Global Navigation Satellite System (GNSS) chipsets, such as Broadcom BCM47755 and Qualcomm Snapdragon 855 embedded, makes instantaneous and cm level real-time kinematic (RTK) positioning possible with Android-based smartphones. In this contribution we investigate the instantaneous single-baseline RTK performance of Samsung Galaxy S20 and Google Pixel 4 (GP4) smartphones with such chipsets, while making use of dual-frequency L1 + L5 Global Positioning System (GPS), E1 + E5a Galileo, L1 + L5 Quasi-Zenith Satellite System (QZSS) and B1 BeiDou Navigation Satellite System (BDS) code and phase observations in Dunedin, New Zealand. The effects of locating the smartphones in an upright and lying down position were evaluated, and we show that the choice of smartphone configuration can affect the positioning performance even in a zero-baseline setup. In particular, we found non-zero mean and linear trends in the double-differenced carrier-phase residuals for one of the smartphone models when lying down, which become absent when in an upright position. This implies that the two assessed smartphones have different antenna gain pattern and antenna sensitivity to interferences. Finally, we demonstrate, for the first time, a near hundred percent (98.7% to 99.9%) instantaneous RTK integer least-squares success rate for one of the smartphone models and cm level positioning precision while using short-baseline experiments with internal and external antennas, respectively.

## 1. Introduction

The use of survey-grade Global Navigation Satellite System (GNSS) receivers and antennas has traditionally been the only solution to achieve fast and high precision positioning. With the emerging GNSSs, such as the BeiDou Navigation Satellite System (BDS), Galileo and the Quasi-Zenith Satellite System (QZSS), low-cost receivers have been proven to be capable to bring a near competitive ambiguity resolution and mm–cm level positioning performance to that of more expensive survey-grade GNSS receivers and antennas [1,2,3,4]. Before 2005, there were fewer than ten smartphone models equipped with GPS [5]. Currently, it is hard to find any smartphone that is without GPS (or GNSS) positioning functionality. There has also been a recent rapid development in small-size and low-cost GNSS smartphone chipsets that adopt dual-frequency, multi-GNSS, code and phase measurements for enhanced positioning precisions [6,7]. 

Several studies [8,9,10,11,12] have highlighted that smartphone antennas are highly sensitive to multipath effects. Pesyna et al. [8] showed by using an auxiliary external antenna connected to a smartphone that it was possible to overcome the multipath sensitivity of the smartphone antennas and subsequently achieved cm level positioning precision. A few other recent studies [12,13] have further suggested that cm level positioning precision can be achieved in a short-baseline model while using the smartphone internal antennas and a Kalman filter to link parameters in time (multi-epoch model). Geng and Li [14] and Gao et al. [15] recently confirmed that there is a presence of carrier phase drifts in some smartphone observation data with an arbitrary phase offset, whereas similar phase offsets and drifts appeared in other earlier and later smartphone positioning studies as well (e.g., [8,9,12,16,17,18]). 

In this contribution, we will assess such anomalies in the phase data and the instantaneous (single-epoch) real-time kinematic (RTK) performance of two Samsung Galaxy S20 and two Google Pixel 4 smartphones, all forming their individual RTK baselines. In our model and different from other studies above is that *all* parameters will be unlinked in time, and the benefit of using the instantaneous RTK model is that it then becomes insensitive to cycle slips. For all analysed baselines we will make use of several hours of data with a one second measurement interval, so that our conclusions will be statistically significant. As a part of our analysis, we will explicitly investigate two setup configurations, namely having the smartphones in an (*i*) upright and (*ii*) lying down position, while tracking GNSS signals from both external and internal antennas. This is motivated by the fact that we want the internal smartphone antennas to be as uninfluenced from the surrounding environment as possible to minimize possibility of signal interference or other effects coming from the poor antenna gain of the smartphones [13].

This contribution is organised as follows. In Section 2, the multi-GNSS RTK functional model is described and in Section 3 the smartphone GNSS data and stochastic model settings are presented. The combined dual-frequency GPS L1 + L5, Galileo E1 + E5a, QZSS L1 + L5 and BDS B1 code and carrier phase observations, are then evaluated in Section 4 by empirical integer least square (ILS) ambiguity success rates (SR) and the obtainable positioning precisions for a zero-baseline experiment. The tracked BDS constellation consists of BDS-2 [19] and BDS-3 satellites [20]. The zero-baseline is formed by placing the smartphones inside a Radio Frequency (RF) shielding box with a re-radiating antenna, which is further connected through an amplifier to an external low-cost antenna. The instantaneous smartphone RTK positioning performance is then assessed, and the characteristics of the double-differenced (DD) carrier-phase and code residuals are analysed while having the smartphones in an upright and lying down position. In this process we will also compare the formal and empirical positioning confidence ellipses and intervals, and analyse whether the data follow a normal distribution. In Section 5, we extend our analysis to investigate the DD phase residuals and the instantaneous RTK positioning performance for a short baseline. In this section, we focus our attention on the performance of external as well as the smartphone internal antennas. Finally, in Section 6, a summary with conclusions is drawn.

## 2. The Multi-GNSS, Dual Frequency, Single-Baseline RTK Functional Model

The functional model we will use assumes two receivers *r* = 1,2 that track *s* = 1, …, *m* GPS, Galileo, QZSS and BDS satellites on *f* frequencies. For the zero- and short-baseline models, the relative ionospheric and tropospheric delays and satellite orbit errors can be assumed negligible. We make use of broadcast ephemerides for satellite orbits and clocks and the instantaneous (single-epoch), linearized DD system of observation equations read,

(1) y=Aa+Bb, 

where *y* is the vector of DD carrier-phase and code observations, *A* is the design matrix of the DD integer ambiguities in vector *a* and *B* corresponds to the design matrix of the real-valued baseline components *b*. The LAMBDA method [21] will be used for full integer ambiguity resolution (IAR) to obtain the precise fixed baseline solution. Provided that the success rate, i.e., the probability of correct integer estimation, is sufficiently high, the baseline will have mm–cm level precision owing to the precise carrier-phase observations. We make use of system-specific reference satellites when performing the between-satellite single-differences. However, it is emphasized that if we would take a common reference satellite on the overlapping frequencies between the systems, it could further strengthen the model provided that the inter-system biases (ISBs) can be calibrated [22,23]. For the stochastic model, an elevation weighting sine-function was used, as employed in RTKlib v2.4.3 [24].

## 3. GNSS Data Collection with Android-Based Smartphones

This section presents the setup configurations of the zero-baseline and short-baselines in upright and lying down positions, and describe the stochastic model settings. In the following sections, we will investigate the effects the setup has on the instantaneous RTK positioning performance and the corresponding DD carrier-phase and code residuals. 

### 3.1. General Setup Configuration with External Antennas

**Figure 1:**
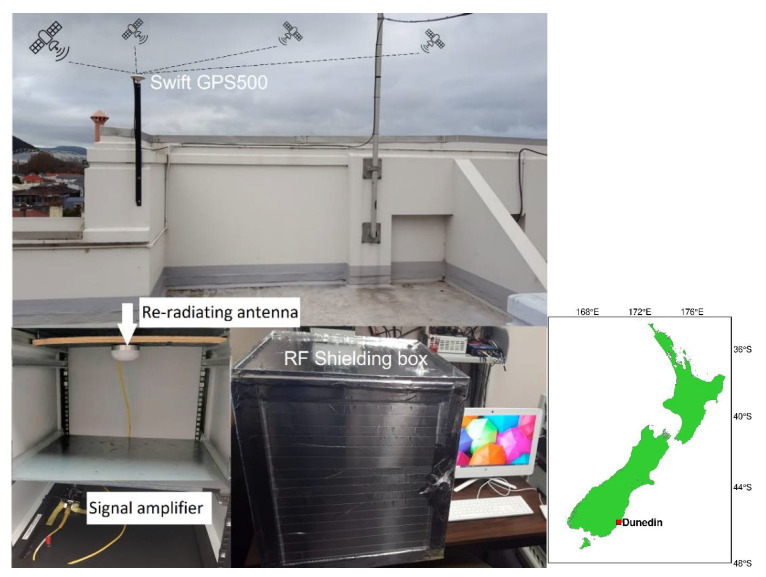
General external antenna setup configuration of S20 and GP4 smartphones that will be benchmarked against survey-grade Trimble NetR9 and low-cost ublox receivers/antennas. All receivers/antennas are placed in a RF shielding box to prevent them from receiving GNSS signals that are not from the re-radiating antenna. The direct GNSS signals are collected from the active low-cost antenna on the rooftop, Swift GPS500, and re-radiated via a passive antenna. A signal amplifier is connected in between the rooftop antenna and re-radiating antenna to reduce the effect of signal attenuation. Inset (right column) depicts the location map of Dunedin, New Zealand.

Figure 1 and Table 1 depict the general external antenna setup to assess the observation quality of the GNSS data collected by two Android-based smartphones, namely, Samsung Galaxy S20 (S20) and Google Pixel 4 (GP4). The duty-cycling settings of all smartphones were disabled during the experiment. Hence, we were able to observe continuous carrier-phase observations without discontinuity [9]. The GP4 and S20 smartphones are able to track dual-frequency GPS L1 + L5, Galileo E1 + E5a, QZSS L1 + L5 and BDS B1 code and phase observations. The receivers and antennas are placed in a radio frequency (RF) shielding box to prevent them from receiving GNSS signals other than from the intended re-radiating antenna. The direct GNSS signals are collected from a rooftop active low-cost antenna, Swift GPS500, which costs a few hundred USDs, and then re-radiated via a passive antenna inside the RF shielding box. A signal amplifier is connected in between the rooftop antenna and re-radiating antenna to reduce the effect of signal attenuation, see Figure 1. The smartphones are also benchmarked against survey-grade Trimble NetR9 receivers + Zephyr-2 antennas and low-cost ublox ZED-F9P receivers + patch antennas that are also placed inside the RF shielding box. The main purpose of this benchmarking is to assure that no GNSS signal leakage is experienced in the RF shielding box that could potentially worsen the RTK performance. Note that L2 rather than L5 observations were tracked by the Trimble and ublox receivers, respectively. For ublox, we did so since, at the time of writing, there was no support for L5 observations, whereas for the Trimble NetR9 receivers we made use of an old firmware version that was possibly not able to track the GPS/QZSS L5 observations [25]. We also note that the analysed smartphones are all restricted (at the time of writing) to L5 rather than L2 observations. 

The Trimble NetR9 and low-cost ublox receivers logged GPS + Galileo + QZSS + BDS measurements at 1-s data interval (cf. Table 1), and the smartphones logged the observations through the Geo++ RINEX Logger vers. 2.1.6. At the time of collecting this data, we found that a maximum timespan of about 17 h and 15 h can be collected for S20 and GP4, respectively. This was limited by a battery-saving mechanism preventing them to operate for longer timespans. The Geo++ RINEX logger developers found a similar issue on e.g., the Xiaomi Mi8 and Huawei P20X smartphones [26]. 

### 3.2. The ‘Upright’ and ‘Lying Down’ Setup Configurations

**Figure 2:**
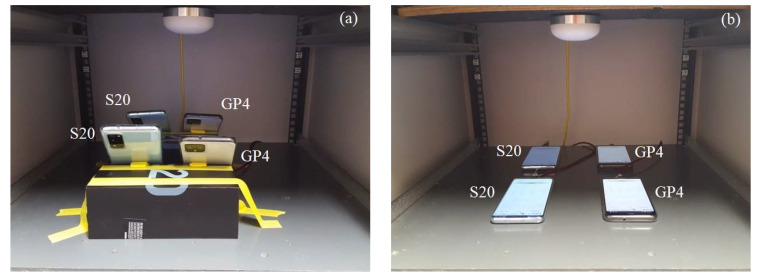
Zero-baseline setup configurations for smartphones in (**a**) upright and (**b**) lying down positions. The re-radiating antenna receives the GNSS signals through the roof-top antenna in Figure 1.

The built-in antennas of the smartphones have been found to be sensitive to poor quality GNSS signals and the surrounding environment [27]. The GNSS signals tracked by the S20 and GP4 smartphones are likely interfered with when placed too close to other objects even inside an RF shielding box. In this contribution we therefore assess the performance of the smartphones under two different setup configurations: (*i*) when the smartphones are in an *upright* position as depicted in Figure 2a and (*ii*) when *lying down* in Figure 2b. As a result of the zero-baseline setup, multipath is largely eliminated. The remaining small multipath effects would mainly be due to the non-simultaneity of sampling between the receivers.

**Figure 3:**
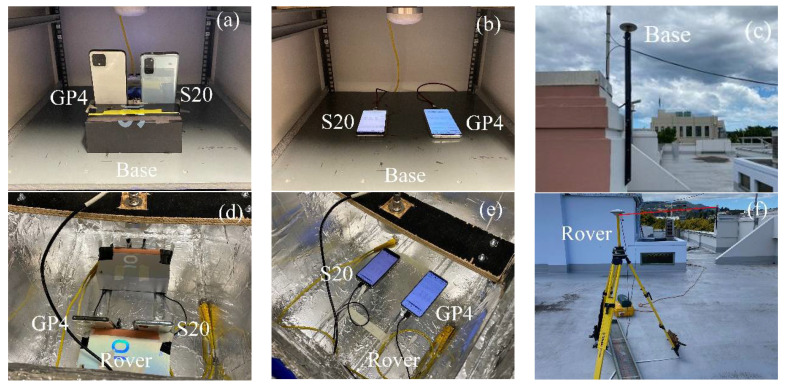
Short-baseline setup configurations with external antennas (**c**,**f**) in upright (**a**,**d**) and lying down (**b**,**e**) positions.

**Figure 4:**
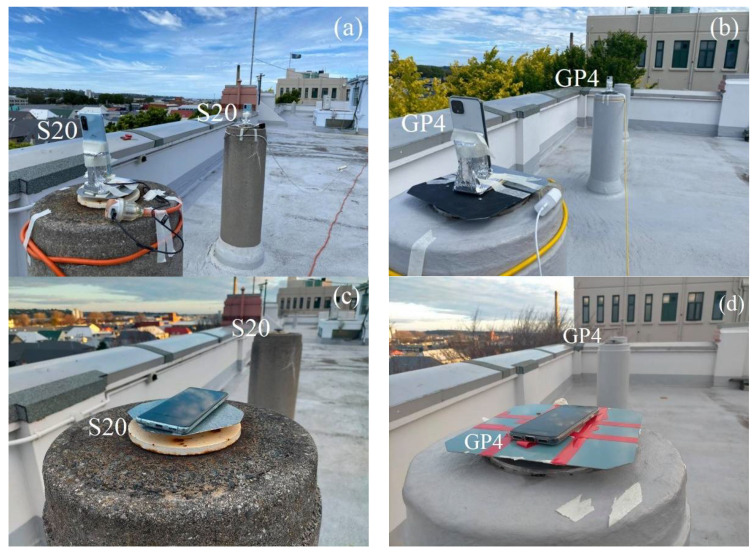
Short-baseline setup configurations with smartphones internal antennas in upright (**a**,**b**) and lying down (**c**,**d**) positions.

In addition to the zero-baselines in Figure 2, we assess the performance of four short-baseline setup configurations, namely with external antennas (*i*) in the upright position (cf. Figure 3a,d) and (*ii*) lying down position (Figure 3b,e). We further show results with internal smartphone antennas in the upright position (Figure 4a,b) and in a lying down position (Figure 4c,d).

### 3.3. Stochastic Model Settings

The stochastic model was then determined by fitting empirical 95 percent confidence ellipses/levels to the formal counterparts, as derived from the corresponding variance covariance (VCV) matrices of the positions. The empirical VCV-matrix is given by the positioning errors as derived by comparing the estimated positions to precise benchmark coordinates, whereas the formal VCV-matrix is given from the mean of all single-epoch formal VCV-matrices of the entire observation span [28]. Independent data were used to formulate the stochastic model for the data to be analysed in the following sections. By making use of a realistic stochastic model, we can assure that all baselines can achieve their best possible ambiguity resolution and positioning performance. 

Table 2 shows the undifferenced and zenith-referenced standard deviations (STDs) employed in the stochastic model, together with a description of all the used setup configurations in Figure 2, Figure 3 and Figure 4. We make use of the same code and phase STDs for each individual frequency/GNSS and an elevation weighting function, as employed in RTKlib [24]. 

We further note that we make use of the upright position stochastic model settings also on that of the lying down positions (cf. Table 3). The dates of the data in Table 2 for the lying down positions are given within square brackets. It will become clear in the following sections that the stochastic model settings in Table 2 are indeed overly optimistic for the data when the smartphones are lying down, which implies that this likely is a suboptimal setup to use.

We note that for all datasets to be compared between the upright and lying down positions, we have taken the GPS and QZSS satellite constellation repeatability period of approximately 23 h and 56 min into account [29]. We also note that for the short-baseline external antenna experiment there is ten days between the datasets (cf. Table 2), allowing us to also take into account the repeatability of the Galileo satellites. Finally, we remark that the common low-noise amplifier (LNA) noise between the receivers is mostly cancelled out in the zero-baseline setup [30].

We can see in Table 2 that the code STDs increase by a factor of five when going from using external antennas in the short-baseline setup, to using internal antennas. For instance, we can see that the GP4 code STD increases from about 1.2 m when using external antennas up to approximately 6.0 m when internal antennas are used, whereas the corresponding S20 code STD increases from about 0.5 m to 2.3 m, respectively. The corresponding GP4 phase STD increases from 2 mm to 4 mm, whereas the S20 phase STD remains the same at 3 mm when going from using external to internal antennas. This increase in code STDs implies that the internal antennas are indeed very sensitive to multipath effects and that the code observations are more affected [31]. We can also see in Table 2 that the phase and code STDs are significantly improved in the zero-baseline setup, since here we can expect any multipath effects to be significantly reduced. 

## 4. Zero-Baseline Instantaneous RTK Positioning with Smartphones in Lying Down and Upright Positions

In this section we examine the positioning performance of the smartphones, S20 and GP4, under the two setup configurations in Figure 2, i.e., the (i) upright position; and (ii) lying down position. The two configurations will be tested in a zero-baseline while using an external antenna (cf. Figure 1). In addition, we will investigate the DD phase and code residuals of each setup. For all our results we make use of a single-epoch RTK model (1) since it is insensitive to cycle slips.

The single-epoch integer least-squares (ILS) success rate (SR) and the empirical positioning mean and STDs (correctly fixed and float positioning errors) for the zero-baseline, in lying down and upright positions, are given in Table 3. This is given together with the mean of the root mean square (RMS) values of the DD phase and code residuals of each GNSS that repeat between datasets (cf. Table 2). The mean was taken as an average RMS over all satellites for each GNSS, and the RMSs for the DD code residuals are shown within square brackets. The number of correctly fixed epochs was determined by the number of epochs where the estimated local North, East and Up coordinate errors are all below or equal 0.05 m. The ILS success rate was then computed as follows,

(2) $$P_{S_{E}}=\frac{\#\;\mathrm{of}\;\mathrm{correctly}\;\mathrm{fixed}\;\mathrm{epochs}}{\mathrm{total}\;\#\;\mathrm{of}\;\mathrm{epochs}}\times 100\%.$$

In other words, the single-epoch ILS SR in Table 3 shows the availability of correctly fixed solutions with mm level positioning STDs. In Table 3, we can see an identical ILS SR of 99.9% for the GP4 smartphones in the lying down and upright positions. We can also see that these results are impressive indeed, since they resemble that of the high-cost Trimble NetR9 and ublox ZED-F9P receivers and antennas with an ILS SR of 99.9% and 100.0%, respectively. However, for the S20 smartphones there is a significant worsening of the performance, where we go from 97.8% to 79.4% ILS SR when the smartphones are upright and in lying down positions, respectively. This despite the fact that the mean number of satellites *increased* from 13 when upright to 17 when lying down. The drop in the number of satellites for when the S20 smartphones are in an upright position implies that there is possibly an outlier rejection threshold now activated to remove low-quality raw GNSS measurements internally in the S20 GNSS logger, similar to the findings in Guo et al. [32] and Paziewski et al. [12] for other Android-smartphones.

If we further inspect the mean RMSs of the DD phase and code residuals in Table 3, we can see a slight increase in RMS values for GP4 when going from an upright to a lying down position for all frequencies (with also the L1 phase QZSS RMS increasing at the sub-mm level). For S20 the code RMSs are all increasing significantly when going from an upright to a lying down position, with the maximum increase in RMS value for L5 GPS increasing from about 1.1 m to 2.0 m. The S20 phase RMSs are also increasing dramatically when going from an upright to a lying down position, where again the largest increase is observed for L5 GPS of 0.005 m to 0.016 m. We also note that all L5 code RMSs are more precise than that of L1 for GP4 and S20, respectively, similar to [33]. 

We can further compare the obtained code and phase RMSs to that of the geodetic (Trimble) and low-cost (ublox) receivers in Table 3, where we can see that all L1 code observations are much less precise for the smartphones. For instance, the L1 code RMS for the GP4 smartphones is about 2.5 m, which can be compared to the Trimble and ublox L1 GPS RMSs of approximately 0.6 m and 0.3 m, respectively. It is worthwhile to note that the ublox ZED-F9P receivers and antennas were also found to have a better L1 code precision than that of Trimble R10 receivers and antennas in Odolinski and Teunissen [4]. Most importantly we can see that the GP4 and S20 smartphones have comparable carrier-phase L1 RMSs and correctly fixed position STDs at the mm level to that of the Trimble and ublox receivers, respectively, when in an upright position. In addition, we can see that the mean positioning errors generally decrease by a few millimetres when going from lying down to an upright position for both smartphone models. Table 3 shows further that the float mean positioning errors and STDs are different between when the smartphones are lying down and are upright, respectively, which is likely related to the earlier mentioned outlier rejection threshold activated differently for the two cases (Guo et al. [32] and Paziewski et al. [12], see also the average number of satellites). We can also note in Table 3 that the mean number of satellites of GP4 is 21 and S20 is 13 when in an upright position, whereas the Trimble and ublox receivers both tracked 25 satellites while L2 observations were collected rather than the L5 GPS. This implies that GP4 have a better redundancy than that of the S20 smartphones, which, as we show in Table 3, positively affects the RTK positioning performance.

**Figure 5:**
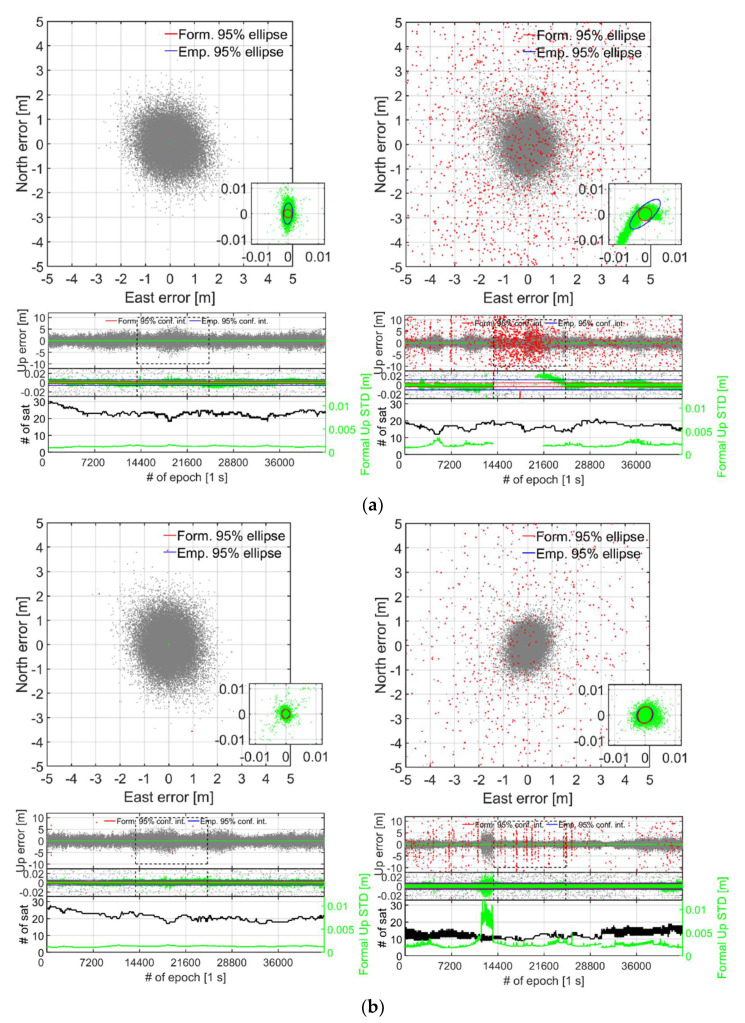
**Zero baseline with smartphones in a (a) lying down and (b) upright position (cf. Figure 2):** Horizontal (N, E) positioning scatter (first row) and corresponding vertical (U) positioning error time-series (second row) for 12 h of data (cf. Table 2) with GP4 (left column) and S20 (right column). In this RTK model GPS + Galileo + QZSS +BDS L1 + L5, E1 + E5a, L1 + L5, B1 observations have been used. A zoom-in window is shown to depict the two orders of magnitude when going from incorrectly fixed (red dots) and ambiguity-float (gray dots), to correctly fixed solutions (green dots). The 95% empirical and formal CIs and ellipses for the correctly fixed solutions are shown as blue and red lines, respectively. The number of visible satellites and the correctly fixed formal up STDs (bottom row) are shown as black and green lines, respectively. The black dashed lines indicate the zoom-in period between epoch 13,800 and 25,000, see further Figure 7, with ILS SRs of 99.9% in both (**a**,**b**) in the left column for GP4 and 31.0% in (**a**) and 97.4% in (**b**) in the right column for S20, respectively. (**a**) Smartphones used in lying down positions: with GP4 (left): ILS SR 99.9% and S20 (right column): 79.4%. (**b**) Smartphones used in upright positions: with GP4 (left): ILS SR 99.9% and S20 (right column): 97.8%.

To further investigate the dramatic drop in the ILS SR for S20 in Table 3 and to evaluate the corresponding RTK positioning performance, Figure 5 depicts the horizontal North/East positioning scatter and vertical Up error time-series for the zero-baseline with the smartphones in the lying down (top panels) and upright position (bottom panels). We employ a zoom-in window to depict the correctly fixed solutions that have mm–cm level positioning precisions and are shown as green dots. The incorrectly fixed and ambiguity-float solutions, shown as red and grey dots, respectively, have meter-level positioning precisions. The GP4 smartphones are shown in the left column and the S20 counterparts in the right column. For all these results, we use 12 h of data with a one second measurement interval, an elevation cut-off angle of 10°, with GPS (L1 + L5), Galileo (E1 + E5a), QZSS (L1 + L5) and BDS (B1) observations, cf. Table 1 and Table 2. The empirical and formal correctly fixed Up-error confidence intervals (CIs) and horizontal error ellipses, depicted as blue and red lines, respectively, have all been determined from the empirical and formal position VCV-matrices. We also depict at the bottom of the Up time-series in Figure 5 the number of satellites for all constellations as black lines, so as to demonstrate the model strengths of the two smartphone models GP4 and S20. On the right hand side *y*-axis we further depict the correctly fixed formal STDs for the Up-component, so as to measure the effect from the receiver-satellite geometry strength on the estimated positioning errors. Note that the lying down position for both GP4 and S20 smartphones achieved an ILS SR of 99.9% and 79.4%, respectively (Figure 5a, cf. Table 3), whereas the corresponding ILS SRs for when the smartphones are in an upright position read 99.9% and 97.8%, respectively (Figure 5b). Finally, we denote epochs 13,800 to 25,000 in Figure 5 with dashed black lines in the Up positioning error panel, since here the S20 smartphones seem particularly affected by the fact that the smartphones are lying down with many incorrectly fixed solutions at the meter level. This particular period will be further investigated in Figure 7.

Figure 5 shows that the S20 smartphones (right column) have a better float precision, as shown as grey dots, compared to that of the GP4 counterparts (left column). The figure further shows that the repeatability of the correctly fixed solutions of the GP4 smartphones, in the left column, are more precise and that GP4 also have less incorrectly fixed solutions (red dots) when compared to that of S20. In particular, we note that less than 3500 out of 11,200 epochs, i.e., an ILS SR of about 31.0%, of the S20 positions were found correctly fixed between epochs 13,800 to 25,000 in Figure 5a, as indicated by the black dashed lines. Figure 5b shows that the S20 results improve when in an upright position with fewer incorrectly fixed solutions. This since the corresponding ILS SRs between epochs 13,800 to 25,000 are now 99.9% and 97.4% for GP4 and S20, respectively, which means that the ILS SR for GP4 remains unchanged but that the ILS SR for S20 has increased by 66.4%. This despite the fact that the mean number of satellites for the same period *decreased* from 16 to 11 satellites when the S20 smartphones were in a lying down position and an upright position, respectively. This implies that the S20 internal smartphone antennas are more sensitive to interferences such as multipath, even inside the RF shielding box (cf. Figure 1). Furthermore, note in Figure 5a,b that the formal STDs of the vertical component of the GP4 is relatively consistent throughout the observation period between the two setups. For S20, however, Figure 5b shows that there is now an increase in the formal correctly fixed STDs (green lines at bottom) when compared to Figure 5a, in particular just before epoch 14,400.

This increase in the formal STDs can be explained by the drastic drop in the number of satellites (cf. Table 3) and the poorer receiver-satellite geometry strength. We found that the vertical dilution of precisions (VDOPs) reached values close to 7 for this period. If we inspect the horizontal positioning scatter in Figure 5a further, we can see that GP4 and S20 in a lying down position setup shows a poor fit both in size and orientation between the empirical (blue lines) and formal (red lines) 95% confidence ellipses, respectively, and a poor fit between the corresponding Up CIs as well. This poor fit implies the use of an overly optimistic stochastic model (cf. Table 2). In contrast, the GP4 and S20 smartphones in an upright position in Figure 5b show a good agreement between the formal and empirical confidence ellipses/intervals. This implies that the stochastic model settings for GP4 and S20 are now realistic. 

**Figure 6:**
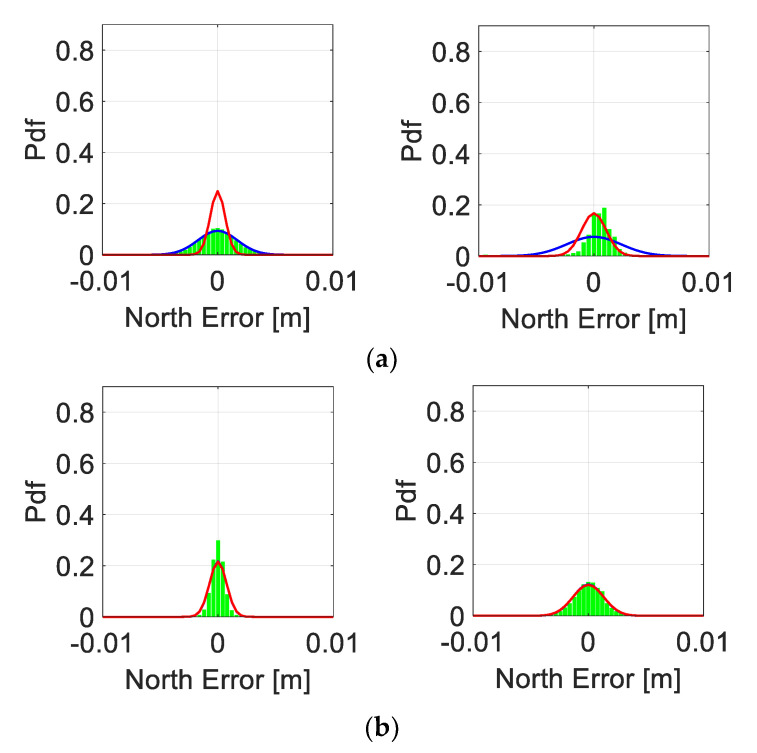
**Zero baseline with smartphones in a (a) lying down and (b) upright position (cf. Figure 2):** North error histograms (green bars) together with their empirical (blue lines) and formal (red lines) PDFs, with GP4 in the left column and S20 in the right column. (**a**) Smartphones used in lying down positions: with GP4 (left) and S20 (right column) using an external antenna. (**b**) Smartphones used in upright positions: with GP4 (left) and S20 (right column) using an external antenna.

To further investigate the distribution of the correctly fixed RTK positioning solutions, we depict in Figure 6 the corresponding North error histograms as green bars. On top of the histograms, we show the corresponding empirical and formal theoretical normal distributions, in terms of their probability density functions (PDFs), depicted as blue and red lines, respectively. These PDFs are based on the empirical mean and empirical and formal STDs of the North positioning errors. The GP4 smartphones are shown in the left column and S20 in the right column, with the top row showing the results when the smartphones are lying down and the bottom row showing the corresponding upright-position results. Finally, we remark that the East and Up positioning errors behave in a similar manner and that their corresponding histograms are thus not shown for brevity.

Figure 6 shows that the empirical normal PDF (blue lines) fit the GP4 histograms very well even when the smartphones are in a lying down position at the top row and left column, but with a poor fit between the formal (red lines) and empirical PDF. However, when the GP4 smartphones are in an upright position, the North errors become more precise, as shown by the more peaked histograms, and the formal and empirical normal PDFs now fit the histograms very well (red lines are on top of the blue lines). On the other hand, for the S20 smartphones we can see an empirical normal PDF that does not fit the histograms of the correctly fixed solutions very well (top row, right column), which implies that the data are not normally distributed when the S20 smartphones are lying down. This improves, however, at the bottom row when the S20 smartphones are in an upright position, where we get more precise North errors with a good fit between the formal and empirical normal PDFs.

**Figure 7:**
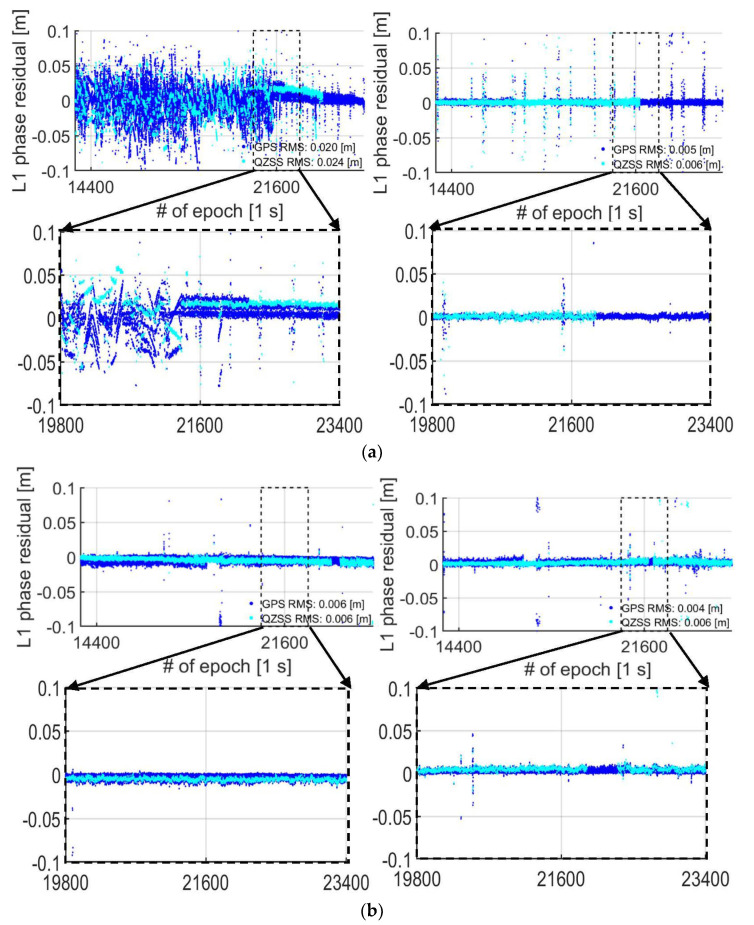
**Zero baseline withS20 in (a) and GP4 in (b), both in a lying down (left column) and upright position (right column):** the DD phase residual time series of the L1 signal (top row) is shown for epoch 13,800 and 25,000 (cf. dashed black lines in Figure 5). The DD phase residuals of GPS are depicted as blue dots and QZSS as cyan dots. The black dashed lines indicate the zoom-in period of the L1 DD phase residuals (bottom row) between the epochs 19,800 and 23,400. (**a**) **S20** smartphones used in the lying down position (left) and upright position (right column) using an external antenna. (**b**) **GP4** smartphones used in the lying down position (left) and upright position (right column) using an external antenna.

To further investigate the problematic period for the S20 smartphones in the zero-baseline setup between epoch 13,800 and 25,000 (cf. dashed black lines in Figure 5), we show in Figure 7a the corresponding L1 GPS and L1 QZSS DD phase residuals with the smartphones in a lying down position in the left column and in an upright position in the right column. The selected zoom-in period at the bottom row depicts a transition period between epochs 19,800 to 23,400 (1 h of data), where it goes from having a poorer RTK positioning performance to a better one (cf. Figure 5). In Figure 7b, we show the corresponding GP4 results. We note that the L5 DD phase residuals show a similar pattern for all our results and are thus not shown for brevity, and that the depicted L1 DD phase residuals are expected to repeat between days as the GPS and QZSS constellation repeatability period was accounted for (cf. Table 2). 

Figure 7a shows, as expected (cf. RMSs in Table 3), that the S20 smartphones obtained larger magnitude of DD phase residuals when they were lying down (left column). In contrast, the S20 smartphones achieved smaller magnitude of DD phase residuals when they are in an upright position (right column), despite having fewer number of satellites (cf. Figure 5). This implies that the smartphone setup has played a significant role in the quality of the phase observations and that the S20 internal antennas seem more sensitive to interferences when the smartphones are lying down. We also remark that there are some large excursions in the DD phase residuals in the top row and right column of Figure 7, when there is also fewer number of satellites, which correspond to incorrectly fixed solutions (cf. Figure 5).

More importantly, in Figure 7a and the left column, we can see that many of the DD phase residuals, when the smartphones are lying down, have a mean different from zero meters and several linear trends present. However, when the smartphones are in an upright position in the right column, we have a zero mean of all residuals and the linear trends become absent. Finally, we can see in Figure 7a that the mean RMS values of the L1 DD phase residuals decrease from 0.020 m and 0.024 m, for GPS and QZSS respectively, to 0.005 m and 0.006 m when the S20 smartphones are in an upright position. 

Pesyna et al. [8] first reported similar linear trends and non-zero-mean DD phase residuals on a Samsung Galaxy S3 for L1 GPS (in their Figure 5) and Humphreys et al. [9] found similar results for a Samsung Galaxy S5 (in their Figure 10). Similar trends were later found in the phase observations in Riley et al. [16] and Håkansson [10] for a Nexus 9 Android-based tablet tracking L1 GPS (and GLONASS) data. Similar trends were also found in, e.g., Geng and Li [14], Wu et al. [17] and Gao et al. [15] for the GNSS observations tracked by Xiaomi Mi8 and in Darugna et al. [34] for a Huawei Mate20X. The common factor between all these studies is that the smartphones were located in a lying down position. We remark, however, that to analyse whether having the smartphones used in the abovementioned studies in an upright position would change their results, would require further investigation outside the scope of our study. 

In comparison to the S20 results in Figure 7a, one can see in Figure 7b that the GP4 performance remains visually more or less unchanged between having the smartphones in a lying down and upright position (left vs. right column). Moreover, there are no apparent linear trends in the DD phase residuals at the bottom row present. However, it is evident that there is a small 2 mm increase in the average RMS values for the GPS L1 DD phase residuals when the smartphones are lying down (whereas the corresponding L1 QZSS RMS remains unchanged). 

## 5. Short-Baseline Instantaneous RTK Performance with Smartphones in Lying Down and Upright Positions

In the previous section, we showed in a zero-baseline setup that the internal smartphone antennas are sensitive to interference from nearby surfaces, even inside a RF shielding box, and that using the upright position rather than having the smartphones lying down will benefit the RTK positioning performance. This is true in particular for the S20 smartphones. We namely observed S20 DD phase residuals with linear trends and non-zero mean values when the smartphones were lying down, which became absent when the smartphones were upright. To further validate whether these effects are also present for short-baselines, we analyse in the following the DD phase residuals and RTK positioning performance with the use of both external and internal antennas (cf. Figure 3 and Figure 4).

### 5.1. Double-Differenced Carrier-Phase Residuals for Short-Baselines Using External Antennas

**Figure 8:**
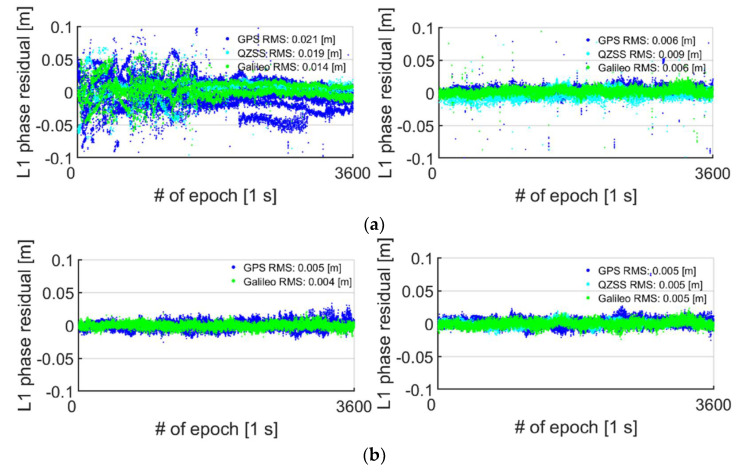
**Short baseline withS20 in (a) and GP4 in (b) using external antennas (cf. Table 2) in lying down (left column) and upright position (right column):** The DD phase residuals for 1 h of data of L1 GPS are depicted as blue, E1 Galileo as green and L1 QZSS as cyan dots. Note that there was only one QZSS satellite in the GP4 dataset in (**b**) and the left column, and thus the corresponding DD phase residuals were not depicted. (**a**) S20 smartphones used in the lying down position (left) and upright position (right column) using external antennas. (**b**) GP4 smartphones used in the lying down position (left) and upright position (right column) using external antennas.

Figure 8 shows the DD phase residuals for L1 GPS, E1 Galileo and L1 QZSS for S20 in the top panels and GP4 in the bottom panels for the first hour of data (out of 8 h, cf. Table 2), while using external antennas in the short-baseline setup configuration (Figure 3). In this analysis, we exclude the BDS DD phase residuals since there is thirty days between the datasets in the left and right column, respectively. This means that only the GPS, QZSS and Galileo constellations are expected to repeat (cf. DOY in Table 2). We do not depict the L5 DD GPS, Galileo and QZSS phase residuals in Figure 8 since they exhibit a similar pattern to L1 and are thus not shown for brevity. Finally, we remark that there was only one QZSS satellite tracked for GP4, when in the lying down position, possibly due to an internal GNSS data logger rejection criterion used, so the corresponding DD phase residuals are not depicted.

In Figure 8a, we can see a poorer RMS in the DD carrier-phase residuals for all three GNSSs when the S20 smartphones are in a lying down position (left column) and when compared to an upright position (right column). We can also see linear trends and some mean of the DD carrier phase residuals that are different to zero meters for S20 in the left column of Figure 8a, similar to the corresponding results in Figure 7a for the zero-baseline setup. Note that the linear trends are absent when the S20 smartphones are upright in the right column of Figure 8a and also have zero mean errors, similar to the results in Figure 7a. For instance, the RMS of the L1 GPS DD phase residuals goes from 0.021 m to 0.006 m when the S20 smartphones are in an upright position. At the same time, we can see that the RMS values for GP4 remain consistent between setups, with the L1 GPS RMS being 0.005 m for both setups, however, with a slight increase from 0.004 m to 0.005 m for E1 Galileo, when having the smartphones upright. This consistency in RMSs implies again that the GP4 smartphone antennas are not as sensitive to interferences when lying down as the S20 smartphones.

### 5.2. Instantaneous RTK Positioning Performance for Short-Baselines with External and Internal Antennas

In Table 4, we summarize the ILS SRs and the empirical positioning mean and STDs (correctly fixed and float positioning errors) for the GP4 and S20 smartphones in the short-baseline experiments while using external (cf. Figure 3) and internal antennas (cf. Figure 4). The ILS SRs are shown for when the smartphones are both in an upright and lying down position, together with the mean number of tracked satellites over the 8 h and 3.5 h data periods for the external and internal antennas, respectively (cf. Table 2). 

Table 4 shows that the ILS SR decreases from 99.2% to 94.9% for S20 when the smartphones go from being in an upright position to lying down while using external antennas. We note that the reduction in ILS SR is a bit smaller for S20 for the short-baseline than in the zero-baseline case in Table 3. This is possibly caused by the shorter amount of data analysed (8 h vs. 12 h, cf. Table 2), as well as the fact that in the zero-baseline case there are more interferences possible since all four smartphones are then put into the *same* RF shielding box (cf. Figure 2). We can also see that when internal antennas are used, we get a poorer ILS SR for S20 of 64.4% when being in an upright position, and 27.5% when lying down. For GP4 we can see in Table 4 (similar to the zero-baseline in Table 3) that the ILS SR remains at 99.9% when external antennas are used and are in upright and lying down position, respectively. 

The situation changes entirely, however, when internal antennas are used. We then go from a GP4 ILS SR of 98.7% to 63.1%, which implies that the GP4 internal antennas also become more sensitive to interferences in the absence of the RF shielding box and the use of external antennas, when more multipath effects are present. Moreover, Table 4 shows that the correctly fixed positioning mean errors are at the mm level and the STDs are always at most one centimetre (in the Up component) when the smartphone are in an upright position rather than lying down. 

**Figure 9:**
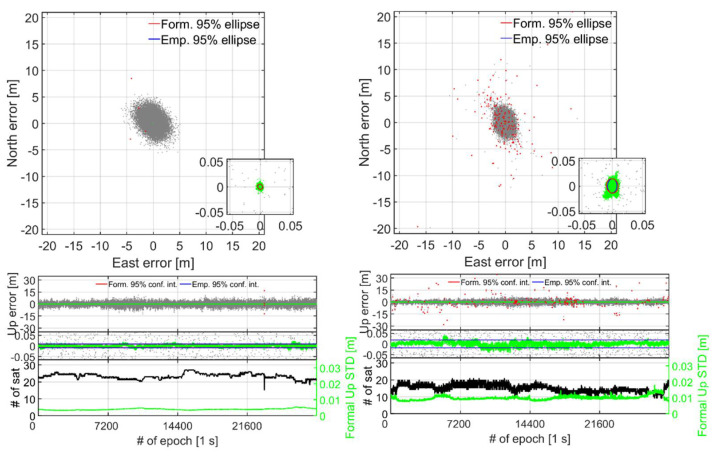
**Short-baseline with external antennas and smartphones in an upright position (cf. Figure 3):** Horizontal (N, E) positioning scatter (first row) and corresponding vertical (U) positioning error time-series (second row) for 8 h of data (cf. Table 2) with GP4 (left column): ILS SR 99.9% and S20 (right column): 99.2%. In this RTK model GPS + Galileo + QZSS + BDS L1 + L5, E1 + E5a, L1 + L5, B1 observations have been used. A zoom-in window is shown to depict the two order of magnitude improvement when going from incorrectly fixed (red dots) and ambiguity-float (grey dots), to correctly fixed solutions (green dots). The 95% empirical and formal confidence CIs and ellipses for the correctly fixed solutions are shown as blue and red lines, respectively. The number of visible satellites and the correctly fixed formal up STDs (bottom row) are shown as black and green lines, respectively.

To further highlight the good instantaneous short-baseline RTK performance of the upright S20 and GP4 smartphones in Table 4 when using external antennas (cf. Figure 3), Figure 9 depicts the corresponding horizontal North/East positioning scatter and vertical Up error time-series (similar to zero-baseline results in Figure 5) for 8 h of data. The GP4 smartphones are again shown in the left column and the S20 counterparts in the right column. We only depict the results when the smartphones are in an upright position for brevity, because the poor fit of the empirical and formal confidence ellipses/intervals and PDFs are similar between these short-baseline results to that of the zero-baseline results in Figure 5 and Figure 6, respectively. 

In Figure 9, we can see a good agreement between the formal and empirical confidence ellipses and intervals denoted as red and blue lines, respectively, which implies that the stochastic model settings in Table 2 are realistic. We can also see that the GP4 smartphones in the left column provides a single-epoch ILS SR of 99.9% even for this short-baseline, which implies that when external antennas are used it can give competitive performance to that of high-cost receivers and antennas with mm–cm level positioning performance. This is similar to the results shown in Odolinski and Teunissen [6] for another Android-based smartphone when connected to low-cost patch antennas. Figure 9 also shows that S20 can achieve an ILS SR 99.2% when external antennas are used and the smartphones are in an upright position. 

Finally, Figure 9 shows that when the correctly fixed Up formal STDs get larger, the corresponding Up errors also get larger for both smartphone models. This happens for instance at the end of the time-series for GP4 in the left column when there is almost a minimum number of tracked satellites over the observation time span, and just before epochs 7200 and 14,400 for S20 in the right column.

**Figure 10:**
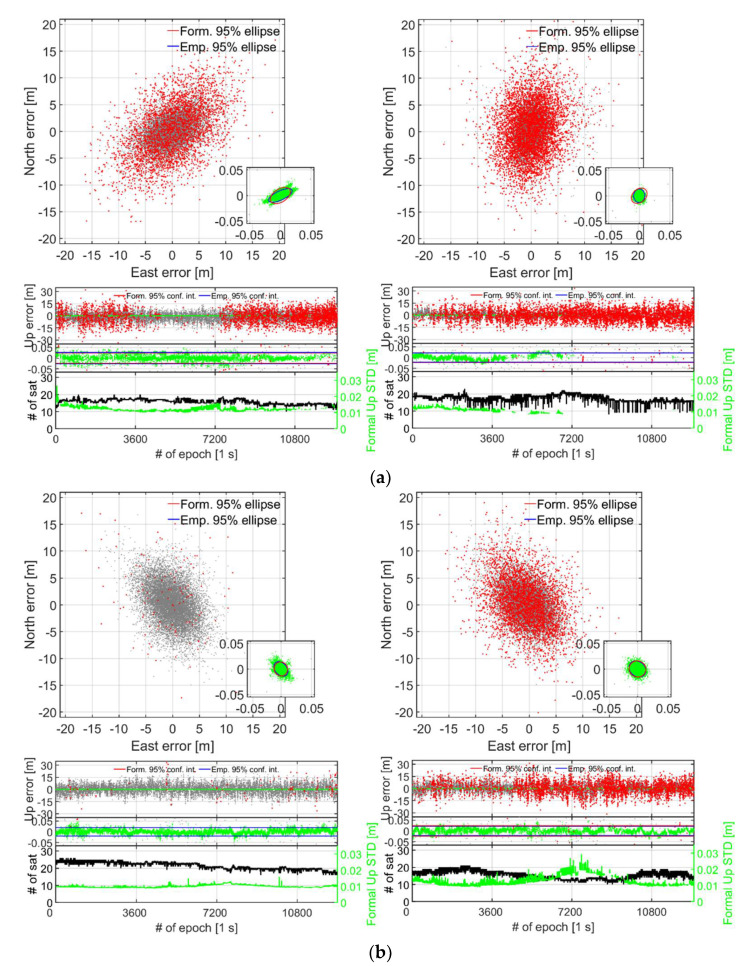
**Short-baseline with internal antennas and smartphones in a (a) lying down and (b) upright position (cf. Figure 4):** Horizontal (N, E) positioning scatter (first row) and corresponding vertical (U) positioning error time-series (second row) for 3.5 h of data (cf. Table 2) with GP4 (left column) and S20 (right column). In all RTK models GPS + Galileo + QZSS + BDS L1 + L5, E1 + E5a, L1 + L5, B1 observations have been used for GP4, except for S20 in (**b**) that did not have more than one QZSS satellite to formulate the DD observations. A zoom-in window is shown to depict the two order of magnitude improvement when going from incorrectly fixed (red dots) and ambiguity-float (grey dots), to correctly fixed solutions (green dots). The 95% empirical and formal CIs and ellipses for the correctly fixed solutions are shown as blue and red lines, respectively. The number of visible satellites and the correctly fixed formal Up STDs (bottom row) are shown as black and green lines, respectively. (**a**) Smartphones used in lying down positions: with GP4 (left): ILS SR 63.1% and S20 (right column): 27.5% (internal antennas). (**b**) Smartphones used in upright positions: with GP4 (left): ILS SR 98.7% and S20 (right column): 64.4% (internal antennas).

To further investigate the instantaneous RTK positioning performance when using internal instead of the external antennas, we show in the top panel of Figure 10 the GP4 and S20 corresponding results when the smartphones are lying down (cf. Figure 4). In the bottom panel (Figure 10b), we also show the corresponding results when the smartphones are in an upright position. 

In Figure 10a, we again see a poor fit of the formal and empirical confidence ellipses and intervals when the smartphones are lying down, whereas when the smartphones are in an upright position, in Figure 10b, they fit each other very well. The S20 smartphones achieve an instantaneous ILS SR of 64.4% when the smartphones are in an upright position (Figure 10b), which decreases to 27.5% when they are lying down (Figure 10a). Moreover, now we can see a decrease in the number of tracked satellites for GP4 when the smartphones are lying down (cf. Table 4). This is possibly due to the internal GP4 GNSS data logger that removes low-quality raw GNSS measurements more consistently with the more multipath-sensitive internal antennas used. Nevertheless, the consequence of having the GP4 smartphones in an upright position increases the ILS SR from 63.1% in Figure 10a and the left column, to 98.7% ILS SR when the GP4 smartphones are in an upright position in Figure 10b. These results are important, as they show that the GP4 smartphones can provide for close to 100% (98.7%) availability of mm–cm level short-baseline positioning precisions, even when internal antennas are used.

## 6. Conclusions

In this contribution, we studied the single-baseline real-time kinematic (RTK) performance while having the smartphones upright and in a lying down position, in a zero-baseline and short-baseline setup, respectively. We made use of radio frequency (RF) shielding boxes and re-radiating antennas to track GNSS signals from external low-cost antennas, consisting of L1, L5 GPS, E1, E5a Galileo, L1, L5 QZSS and B1 BDS code and carrier-phase observations, while using two Google Pixel 4 and two Samsung Galaxy S20 smartphones. We assessed their instantaneous (single-epoch) RTK performance and the double-differenced (DD) carrier-phase and code residuals, while also using the internal smartphone antennas. We found that when having the smartphones lying down, the RTK positioning performance, for S20 in particular, will deteriorate. This was also true for the GP4 smartphones when using internal antennas in a short-baseline experiment. 

Our zero-baseline instantaneous RTK evaluation consisted of a formal and empirical analysis of the smartphones, in an upright and lying down position, while using 12 h of data with a one second measurement interval. When the smartphones were lying down, we found that the S20 smartphones seem particularly affected by the setup configuration with many incorrectly fixed solutions at the meter-level precision. The integer least-squares (ILS) success-rate (SR), i.e., the probability of correct integer estimation, while using full ambiguity resolution, improved significantly when having the S20 smartphones in an upright position (cf. Figure 5). The GP4 smartphones showed more or less no ILS SR improvement between the two zero-baseline setup configurations. 

However, the formal and empirical positioning confidence ellipses/intervals and probability density functions (PDFs), respectively, showed a contrast difference from each other when all the smartphone models were lying down. This implies that the GP4 antennas were also affected by interferences from the smartphone configuration.

The vital influence of the setup configuration was also reflected in the observation data quality, in which linear trends were found to be present in the S20 DD phase residuals while the phones were lying down. The mean root mean square (RMS) values of these DD phase residuals were higher when the smartphones were in a lying down position rather than upright. This was also particularly pronounced in the S20 smartphones for the DD code residuals. It is worth noting that in these zero-baseline experiments, the multipath from the external antennas is expected to be significantly reduced and any remaining small multipath effects would mainly be due to the non-simultaneity of sampling between the receivers. Nevertheless, we found that the RMSs of the GPS and QZSS DD phase residuals resembles those of Trimble NetR9 and ublox ZED-F9P receivers in the zero-baseline setup, whereas the corresponding RMSs of the L1 DD code residuals were several times larger for both smartphone models (cf. Table 3). Finally, we found that both smartphone models obtained correctly fixed positioning error STDs that also resemble that of the Trimble NetR9 and ublox ZED-F9P receivers when in an upright position.

In the short-baseline instantaneous RTK results, when multipath from the external/internal antennas is expected to be present, we found consistent worsening in the results when the smartphones were lying down (similar to that of the zero-baseline analysis). Most importantly we found encouraging instantaneous, short-baseline, cm level RTK positioning results for the GP4 and S20 smartphones when observing signals from external antennas, with ILS SR close to 100% (99.9%) for GP4 and 99.2% for S20, respectively, while using 8 h of data. When use was made of internal antennas and having the smartphones in an upright position, we achieved a corresponding ILS SR of 98.7% and 64.4% for GP4 and S20, respectively, based on 3.5 h of data. Overall, we concluded that both smartphone models were influenced by having them in a lying down position, and we suggest that future studies will need to also consider the setup configuration of the smartphones. 

## Figures and Tables

**Table 1:** Site identity, receivers and the GNSS signals used in this study.

Site ID	Receiver	Chipset	Constellations	Frequencies
SGD1	Samsung Galaxy S20	Broadcom BCM47755	GPS, Galileo, QZSS, BDS	L1 + L5, E1 + E5a, L1 + L5, B1
SGD2				
GPD1	Google Pixel 4	Qualcomm Snapdragon 855 embedded	GPS, Galileo, QZSS, BDS	L1 + L5, E1 + E5a, L1 + L5, B1
GPD2				
UBX1	μBlox	F9P	GPS, Galileo, QZSS, BDS	L1 + L2, E1 + E5b, L1 + L2, B1 + B2
UBX2				
TRD1	Trimble NetR9	MaxwellTM 6	GPS, Galileo, QZSS, BDS	L1 + L2, E1 + E5b, L1 + L2, B1 + B2 + B3
TRD2				

**Table 2:** The zenith-referenced and undifferenced code/phase STDs for zero- and short-baseline setup configurations, where we make use of the same STD for all frequencies (L1/L5/E1/E5a/B1) and GNSS for each smartphone setup. In the last column [within square brackets we depict the day of year (DOY) and Coordinated Universal Time (UTC) time for when the smartphones were in a lying down position].

Baselines	Phase STD (m)	Code STD (m)	Description	DOY, Hours of Data, hh:mm:ss UTC
Zero-baseline external antenna:				
GPD1–GPD2	0.001	0.457	Smartphones in upright (Figure 2a) and lying down (Figure 2b) positions.	2020 (12 h): 305, 01:16:00–13:15:59 [298, 01:44:00–13:43:59]
SGD1–SGD2	0.001	0.175		
Short-baseline external antennas:				
GPD1-GPD2	0.002	1.198	Smartphones in upright (Figure 3a,d) and lying down (Figure 3b,e) positions.	2021 (8 h): 228, 13:35:00–21:34:59 [258, 11:35:00–19:34:59]
SGD1–SGD2	0.003	0.485		
Short-baseline internal antennas:				
GPD1-GPD2	0.004	5.997	Smartphones in upright (Figure 4a,b) and lying down (Figure 4c,d) positions.	2020 (3.5 h): 344–345, 21:10:00– 00:41:59 2021 (3.5 h): [261, 02:18:00–05:49:00]
SGD1–SGD2	0.003	2.327		

**Table 3:** Empirical ILS SR for instantaneous zero-baseline dual-frequency RTK positioning and 12 h of data (cf. Figure 2). The instantaneous (single-epoch) empirical mean and STDs (East, North and Up) of the correctly fixed and float positioning errors are also shown. The mean RMSs of the DD phase (code within square bracket) residuals are also depicted in the last column for GPS and QZSS that repeat between the datasets (cf. Table 2).

Baselines	Setup	ILS SR (%)	Avg. # of Sat.				Mean RMS of DD Phase [Code] Residuals (m)					
				Mean ± STDs (m)			GPS			QZSS		
					Correctly Fixed	Float	L1	L2	L5	L1	L2	L5
GPD1–GPD2	upright	99.9	21	E	0.000 ± 0.001	−0.014 ± 0.580	0.003 [2.479]	-	0.003 [0.243]	0.004 [2.319]	-	0.003 [0.248]
				N	0.000 ± 0.001	0.004 ± 0.702						
				U	0.004 ± 0.003	0.006 ± 1.429						
GPD1–GPD2	lying down	99.9	23	E	0.000 ± 0.001	0.006 ± 0.599	0.005 [2.543]	-	0.004 [0.312]	0.004 [2.323]	-	0.004 [0.274]
				N	0.000 ± 0.002	−0.005 ± 0.627						
				U	−0.008 ± 0.003	−0.030 ± 1.429						
SGD1–SGD2	upright	97.8	13	E	0.000 ± 0.001	0.005 ± 0.338	0.005 [1.401]	-	0.005 [1.119]	0.005 [1.043]	-	0.006 [0.932]
				N	0.000 ± 0.001	−0.011 ± 0.411						
				U	0.001 ± 0.003	−0.017 ± 0.920						
SGD1–SGD2	lying down	79.4	17	E	0.000 ± 0.002	−0.019 ± 0.473	0.012 [2.053]	-	0.016 [2.028]	0.013 [1.735]	-	0.013 [1.644]
				N	0.001 ± 0.002	0.001 ± 0.555						
				U	−0.004 ± 0.006	−0.017 ± 1.251						
TRM1–TRM2	upright	99.9	25	E	0.000 ± 0.001	0.004 ± 0.215	0.004 [0.586]	0.003 [0.525]	-	0.006 [0.569]	0.002 [0.404]	-
				N	0.000 ± 0.001	0.000 ± 0.238						
				U	0.000 ± 0.002	0.045 ± 0.576						
UBX1–UBX2	upright	100.0	25	E	0.000 ± 0.001	−0.027 ± 0.158	0.005 [0.303]	0.005 [1.022]	-	0.004 [0.441]	0.004 [0.843]	-
				N	0.000 ± 0.001	0.032 ± 0.198						
				U	−0.010 ± 0.002	0.046 ± 0.352						

**Note:** The dashes indicate when the L2/L5 DD phase/code residuals are not tracked in our zero-baseline setup. Note that the Galileo MEO satellites have a repeatability of every tenth sidereal day and the BDS MEO satellites every eleventh day, and these GNSSs are thus excluded from the comparison. The mean RMS values of the DD phase and code residuals are determined based on the period stated in Table 2. Finally, Avg. # of sat. denotes the average number of satellites over the entire observation time-span.

**Table 4:** Empirical ILS SRs, mean and STDs (East, North and Up) of correctly fixed and float positioning errors for instantaneous short-baseline dual-frequency RTK positioning, with 8 h and 3.5 h of data used for the external and internal antennas, respectively (cf. Table 2). Average number of satellites is denoted by Avg. # of Sat.

Baselines	Antenna Type	Setup	ILS SR (%)	Avg. # of Sat.	Mean ± STDs (m)		
						Correctly Fixed	Float
GPD1–GPD2	External	upright	99.9	24	E	−0.001 ± 0.002	−0.105 ± 1.200
					N	−0.000 ± 0.002	0.375 ± 1.294
					U	−0.000 ± 0.005	−0.121 ± 2.567
		lying down	99.9	20	E	−0.001 ± 0.002	−0.016 ± 1.243
					N	−0.001 ± 0.002	0.238 ± 1.312
					U	−0.002 ± 0.005	0.048 ± 2.644
	Internal	upright	98.7	21	E	0.001 ± 0.005	−0.222 ± 2.977
					N	−0.001 ± 0.006	0.209 ± 3.483
					U	0.001 ± 0.010	0.370 ± 5.518
		lying down	63.1	16	E	−0.002 ± 0.008	−0.098 ± 2.909
					N	−0.000 ± 0.005	0.086 ± 2.516
					U	−0.002 ± 0.013	−0.907 ± 4.282
SGD1–SGD2	External	upright	99.2	15	E	0.000 ± 0.003	−0.031 ± 0.729
					N	−0.000 ± 0.006	0.091 ± 1.013
					U	−0.005 ± 0.009	−0.050 ± 1.730
		lying down	94.9	20	E	0.006 ± 0.006	−0.037 ± 0.707
					N	0.002 ± 0.007	0.043 ± 0.939
					U	0.001 ± 0.010	−0.124 ± 1.874
	Internal	upright	64.4	16	E	0.001 ± 0.005	−0.222 ± 2.977
					N	−0.001 ± 0.006	0.210 ± 3.483
					U	−0.001 ± 0.010	0.274 ± 4.677
		lying down	27.5	17	E	0.001 ± 0.004	−0.157 ± 1.531
					N	0.002 ± 0.005	0.103 ± 2.499
					U	−0.006 ± 0.011	−0.214 ± 3.384

## Data Availability

The broadcast ephemerides were used for satellite orbits and clocks. The Google Pixel 4 and Samsung Galaxy S20 smartphones observation data are stored at the University of Otago–the School of Surveying data facilities and can be made available upon request by contacting the corresponding author, C.Y. by email.
